# Full fingerprint hyperspectral imaging of prostate cancer tissue microarrays within clinical timeframes using quantum cascade laser microscopy†

**DOI:** 10.1039/d5an00046g

**Published:** 2025-03-10

**Authors:** Dougal Ferguson, Niels Kroeger-Lui, Domenic Dreisbach, Claire A. Hart, Diego F. Sanchez, Pedro Oliveira, Mick Brown, Noel Clarke, Ashwin Sachdeva, Peter Gardner

**Affiliations:** a Photon Science Institute, University of Manchester Oxford Road Manchester M13 9PL UK peter.gardner@manchester.ac.uk; b Department of Chemical Engineering, School of Engineering, University of Manchester Oxford Road Manchester M13 9PL UK; c Bruker Optics GmbH & Co. KG Ettlingen Germany; d Division of Cancer Sciences, University of Manchester UK; e Cancer Research UK Manchester Institute Wilmslow Road Manchester M20 4GJ UK; f Department of Pathology, The Christie Hospital NHS Foundation Trust UK; g Department of Surgery, The Christie Hospital NHS Foundation Trust UK; h Department of Urology, Salford Royal Hospital UK

## Abstract

One of the major limitations for clinical applications of infrared spectroscopic imaging modalities is the acquisition time required to obtain reasonable images of tissues with high spatial resolution and good signal-to-noise ratio (SNR). The time to acquire a reasonable signal to noise spectroscopic scan of a standard microscope slide region of tissue can take many hours. As a trade-off, systems can allow for discrete wavenumber acquisitions, sacrificing potentially vital chemical bands in order to reach specific acquisition targets. Recent instrumentation developments now allow for the full fingerprint imaging of entire microscope slides in under 30 minutes, enabling rapid, high quality spectroscopic imaging of tissues within clinical timeframes without sacrificing frequency bands. Here we compare the data from a novel QCL microscope to an FTIR microscope covering multiple aspects of spectroscopic imaging of a large, clinically relevant, prostate cancer tissue cohort (*N* = 1281). Comparisons of hyperspectral data acquisition quality in both achieved signal to noise and image contrast alongside the capacity for unsupervised and supervised modelling of tissue constituents are reported. We conclude that it is now possible to collect full fingerprint spectra and derive clinically relevant data in a timeframe suitable for translation into the pathology laboratory without the need to resort to discrete frequency imaging with subsequent loss of information.

## Abstract 2

A QCL microscope is compared to a state-of-the-art FTIR system and is found to capture prostate tissue data twenty times faster, with greater signal to noise, and improved tissue classification, paving the way for clinical adoption.

## Graphical abstract

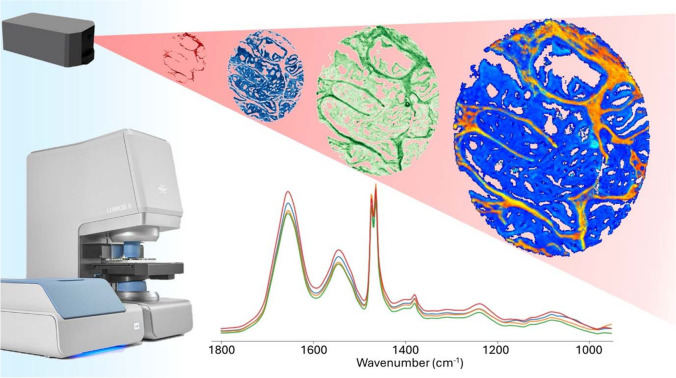

## Introduction

Prostate cancer (PCa) is the most common cause of cancer in males within the United Kingdom, with approximately 50 000 new diagnoses and 12 000 deaths each year.^1^ Unfortunately, PCa is ubiquitous in an aging population, meaning that the number of cases is growing year on year, thus putting an ever-increasing strain on the UK healthcare system. This problem is confounded by the number of UK pathologists taking retirement and a concomitant lack of training and recruitment to the service. This has prompted Cancer Research UK to report “Immediate action is needed to avert a crisis in pathology capacity and ensure we have a service that is fit for the future”.^2^ The increased use of technology, particularly the recent use of AI, is seen a part of the solution. However, although digital pathology and the automated analysis of whole slide images offers some key benefits to the pathologist, the information content remains the same whether analysed by computer or by eye. Infrared spectroscopic analysis of tissue can potentially augment current histopathological practice since additional information is obtained in the form of a chemical fingerprint, indicative of tissue type and disease state.

Although it was over 70 years ago that infrared spectroscopy was first applied to a biomedical specimen,^3,4^ and the first infrared reflecting microscopes were developed,^5^ the low sensitivity coupled with a lack of fundamental understanding of the interaction of infrared radiation with a chemically heterogeneous and morphologically complex sample, meant that it was another 40–50 years before any significant progress was made.^6^ The advent and widespread adoption of FTIR revolutionised infrared spectroscopy, with the Michelson interferometer giving both a multiplex and significant throughput advantage over scanning instruments. However, in the context of conventional infrared microscopy, this advantage is partially negated by the fact that the amount of light impinging on the sample through a narrow microscope aperture, is significantly attenuated, leading to poor SNR at high spatial resolution. This led to the wide spread use of ultra-bright synchrotron sources that could obtain diffraction limited spatial resolution with reasonable SNR.^7–10^ Even so, mapping a tissue sample, by raster scanning, meant that data acquisition times were prohibitively long for clinical samples. Thus the majority of biomedical studies were related to the analysis of single cells.^11–17^ It was really the coupling of infrared microscopes with large area focal plane array (FPA) detectors that facilitated the wider use of standard benchtop instrumentation for tissue imaging research.^18,19^ As a result Infrared spectroscopy has become an appealing modality for molecular diagnostic and prognostic research within the clinical space.^20–25^ Previous works have shown the capacity for IR spectroscopy to be used across multiple facets of biomedical research: from histopathological recognition,^19,26,27^ to digital histopathology,^24,28,29^ to classification of cancerous tissues for diagnostic and prognostic applications.^8,30^

Specifically for prostate cancer, it has been shown that infrared analysis has been successfully employed with the ability to distinguish cell-types, Gleason grade and stage, and has the potential to predict the presence of prostatic adenocarcinoma.^26,31–34^ However, despite the advances in instrumentation and the ability to significantly augment the diagnostic process, FTIR imaging is still too slow for adoption in a pathology laboratory for routine biopsy analysis.

For measuring using a Fourier-Transform Infrared Spectroscopy (FTIR) modality, trade-off rules allow for faster acquisitions at the sacrifice of spectral quality (either through lowering of scan co-additions, thus reducing SNR, or decreasing spectral resolution resulting in loss of information). However, even using large (128 × 128) focal plane array detectors, whole slide imaging (25 × 75 mm^2^) can still take many hours.^35^ The use of tuneable mid infrared Quantum Cascade Laser (QCL) systems offer much faster acquisition times and significant work has been carried out in this field.^36–42^ In most cases the faster data acquisition is due to discrete frequency imaging, at the expense of sacrificing the number of wavenumbers at which measurements are made.^43,44^ Previous QCL microscopes that utilised focal-plane array detectors to image full fingerprint wavenumber regions would still take upwards of 13 hours to image a region covering a standard microscope slide.^41,45^ However, recent advancements in QCL spectroscopic instrumentation can enable scanning of this region within an hour. A modern QCL-based imaging IR microscope can take a hyperspectral image covering a 320 mm^2^ region (an approximate size of a moderate tissue TMA) scanned at 2 cm^−1^ spacing with a nominal pixel size of 4.3 μm in around 53 minutes (up to the limit of the instrument's working distance).^46^ The novel LUMOS II ILIM system (Bruker Optics GmbH & Co KG) improves further on this acquisition time, capturing the same region at identical spacing and pixel size in under 22 minutes. The improvements of the QCL modality is not without issues, as unlike globar source spectrometers, QCL systems have high degrees of temporal and spatial coherence (called the coherence effect).^47^ As such, the results of these systems must be compared against those acquired with well-established commercial FTIR infrared microscopes.

In this paper we report on a comparison of a prostate cancer tissue microarray comprising of 1281 representative tissue cores from 183 patients imaged on one of the most commonly used FTIR instrument with a large FPA namely a Cary 620 FTIR microscope (Agilent Technologies Inc.)^48–51^ and a new state-of-the–art QCL system namely the LUMOS II ILIM (Bruker Optics GmbH & Co. KG), herein referred to as FTIR and QCL respectively. Comparisons will cover instrument setups, the acquisition of hyperspectral data including analysis of image contrast and SNR, and the performance of supervised classification techniques applied to the data. To ensure fair comparison, data treatments were designed to follow the same steps (where possible) with sample annotations being completed on the same set of training cores across similar regions and components of tissue.

## Patients and methods

### Tissue collection

Human prostate tissue samples were obtained from the Manchester Cancer Research Cancer Biobank (10_NOCL_02) from tissue collected following ethical approval by the South Manchester Research Ethics Committee (Ref: 22/NW/0237). Formalin-fixed paraffin-embedded prostate tissue samples from patients undergoing transurethral resection of the prostate (TURP) or transrectal ultrasound guided needle core biopsies (TRUSBx) between 1994 and 2004 were used to construct tissue micro arrays (TMAs). Clinical characteristics of the patient cohort are tabulated in the ESI (Table 1†). Serial tissue sections were cut at 5 μm for each TMA block, with one section being H&E-stained and imaged, with the remainder loaded onto calcium fluoride (CaF_2_) slides for spectral imaging.

### FTIR data acquisition

As indicated in Table 1, FTIR scans of the tissues were acquired using an Agilent Cary 670-IR spectrometer coupled to an Agilent Cary 620-IR imaging microscope, equipped with a liquid nitrogen cooled mercury cadmium telluride (MCT) focal plane array (FPA) detector with 128 × 128 detector elements. A 15× Cassegrain microscope objective was used producing a field-of-view measuring 704 × 704 μm^2^, with each pixel measuring 5.5 × 5.5 μm^2^. A sealable enclosure covered sample stage and optics, providing a continuous supply of dry air. Data were collected with a humidity level <1% to remove any water vapour from the compartment that could have been recorded as part of the spectrum. Background scans, collected from a section of clean paraffin-free calcium fluoride (CaF_2_), consisted of 256 co-additions at a spectral resolution of 5 cm^−1^. For tissue scans images were obtained as a mosaic of multiple tiles, each with 8 co-added scans. Blackmann-Harris interferogram apodisation was used with two levels of zero filling, with a spectral range of 900 to 3800 cm^−1^.

**Table 1:** Key setup information of FTIR and QCL spectrometers used to acquire hyperspectral datasets

	FTIR	QCL
Objective	15×	4×
Numerical aperture (NA)	0.6	0.6
FPA size (pixels)	128 × 128	520 × 480
Nominal pixel size	5.5 μm	4.3 μm
FOV	704 × 704 μm^2^	2210 × 2040 μm^2^
Source	Globar	QCL
Background co-additions	256	1
Scan co-additions	8	1
Spectral resolution	5 cm^−1^	4 cm^−1^
Time to scan (∼28 × 18 cm^2^)	480 minutes	25 minutes

### QCL data acquisition

All experiments were performed in transmission mode on a Bruker LUMOS II ILIM, which is a quantum-cascade-laser microscope equipped with a 520 × 480 focal plane array (FPA) room temperature microbolometer and a refractive 4× IR objective lens (0.6 NA). This combination enables high-speed IR imaging with 2.2 × 2 mm^2^ field of view (FOV) and a nominal pixel size of 4.3 × 4.3 μm^2^. A hardware-based, patented coherence reduction mechanism enables the acquisition of sharp IR images. The instrumental optics were purged with dry air and despite the sample compartment having an open design, no significant water vapour bands were detected in the recorded spectra. A single background scan was collected from an area of clean paraffin-free calcium fluoride (CaF_2_) for each slide, respectively. Full spectra were recorded in sweep scan mode within a spectral range of 1800 to 950 cm^−1^ using a spectral sampling interval of 2 cm^−1^ without co-added scans (*i.e.*, one scan per sampling area). As a result, 249 600 spectra were acquired simultaneously for each sampling area (2.2 × 2 mm^2^) within 16 s, resulting into an IR imaging speed of 16.8 mm^2^ per minute. The total measurement time (*i.e.*, from inserting the sample to completing data acquisition) for one TMA slide was about 25 minutes.

### Data pre-processing

All spectra captured across both modalities underwent the following pre-processing steps where applicable, performed in Python (version 3.9) using the PyIR toolkit in Spyder.^52,53^ Many pre-processing protocols were considered based on other works in classification of cancerous tissues.^54^ Greater detail on the implementation of these pre-processing steps has been provided and discussed in prior works.^54^ Pre-processing protocols for both modalities are as follows:

### FTIR quality control

Quality control consisted of integrating the amide I peak region of 1600–1700 cm^−1^, with any integrated peak values under 2 being discarded. This is done to ensure mixed pixels with significant contribution from wax or calcium fluoride are removed. To ensure comparability, the FTIR data is then truncated to the 950–1800 cm^−1^ region. No additional quality control steps were completed such as anomaly detection.^55^

### QCL quality control

Quality control consisted of integrating the amide I peak region of 1600–1700 cm^−1^, with any integrated peak values under 2 being discarded. A tissue filter binary mask is then generated from this thresholding.

Objects within this binary mask are then identified, with any object smaller than 100 pixels being discarded. This step removes any tissue debris or small interferents from areas surrounding the tissue cores that passed the initial thresholding check.

### Common pre-processing steps

Following quality control, spectra from both FTIR and QCL systems underwent the following processing steps:

- Linear baseline subtraction.

- Removal of the paraffin peak region of 1360–1490 cm^−1^.

- Vector normalisation.

- Minimum noise fraction denoising (with 30 bands for reconstruction).^56^

- 2^nd^ derivative conversion using a Savitsky–Golay (SG) filter with a 21 wavenumber window size and 5^th^ order fitted polynomial.^57^

## Results

### Comparing SNR and spatial resolving power of measurements

**Fig. 1:**
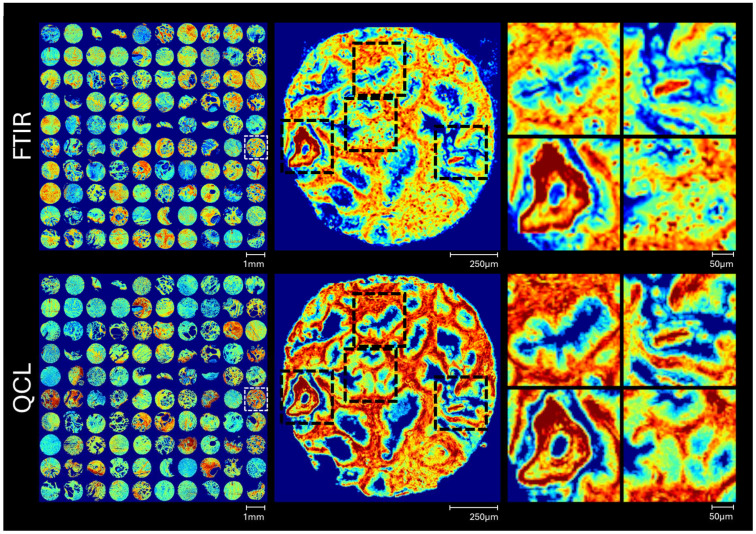
Comparison of amide I peak integration (1600–1700 cm^−1^) images for 100 cores imaged on d FTIR and QCL instruments (left). A single core is highlighted (white dotted lines, left) in greater detail (centre), with four selections from within the core also plotted for comparison of image quality (right).

The first level of comparison can be conducted at the point of data collection (without pre-processing). Image quality such as image contrast and image resolution can be compared through integration of the amide I peak (1600–1700 cm^−1^). This peak is a strong absorber across both modalities so is apt for comparison. A comparison of image quality is plotted in Fig. 1. The improved nominal pixel size of the QCL system appears to result in better contrast in the images, while also decreasing the number of mixed pixels where pixel contribution comes from paraffin or sample substrate.

While SNR values could not be directly calculated for both modalities for comparison, the root mean squared deviation (RMSD) across a small wavenumber window (1780–1800 cm^−1^) is a sufficient surrogate for SNR comparisons. This value is calculated as the square root of the mean squared deviation of absorbance values (*x*_*i*_) from the mean absorbance (x̅) within the spectral window (across N wavenumbers), as expressed in eqn (1). This value represents the change in absorbance values measured in a spectral region with no expected chemical peaks, providing a measure of how much the signal deviates in this spectrally silent region. The RMSD values were calculated using 809 000 spectra for the FTIR data, and 995 000 spectra for the QCL data, and show an improved RMSD for the QCL data (Table 2).1
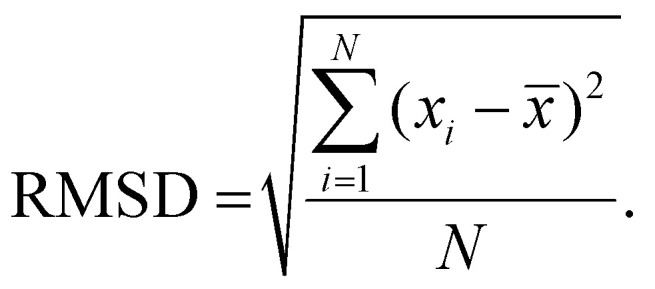


**Table 2:** Root Mean Squared Deviation (RMSD) of datapoints between 1780–1800 cm^−1^ for both FTIR and QCL datasets

RMSD (1780–1800 cm^−1^)	FTIR	QCL
Median	1.50 × 10^−3^	4.45 × 10^−6^
iqr_25_	1.09 × 10^−3^	3.00 × 10^−6^
iqr_75_	2.10 × 10^−3^	6.74 × 10^−6^

**Fig. 2:**
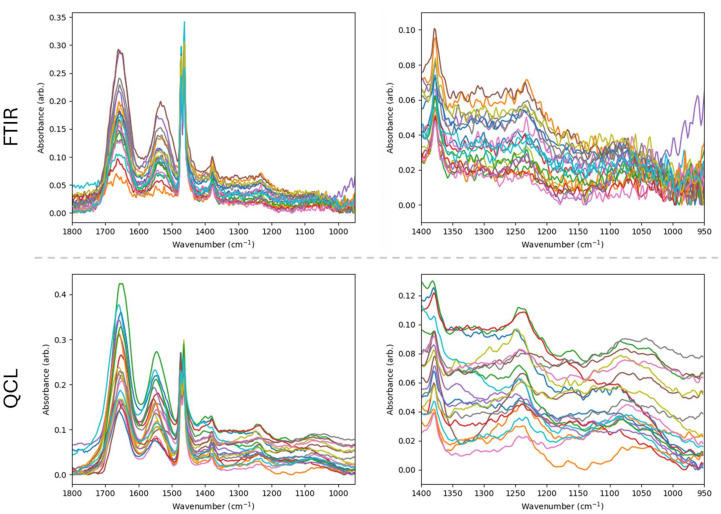
Comparison of 20 randomly sampled raw (unprocessed) spectra collected on FTIR (top) and QCL (bottom) systems. Plots are generated for full fingerprint regions (1800–1000 cm^−1^) and lower wavenumber regions (1400–1000 cm^−1^).

**Fig. 3:**
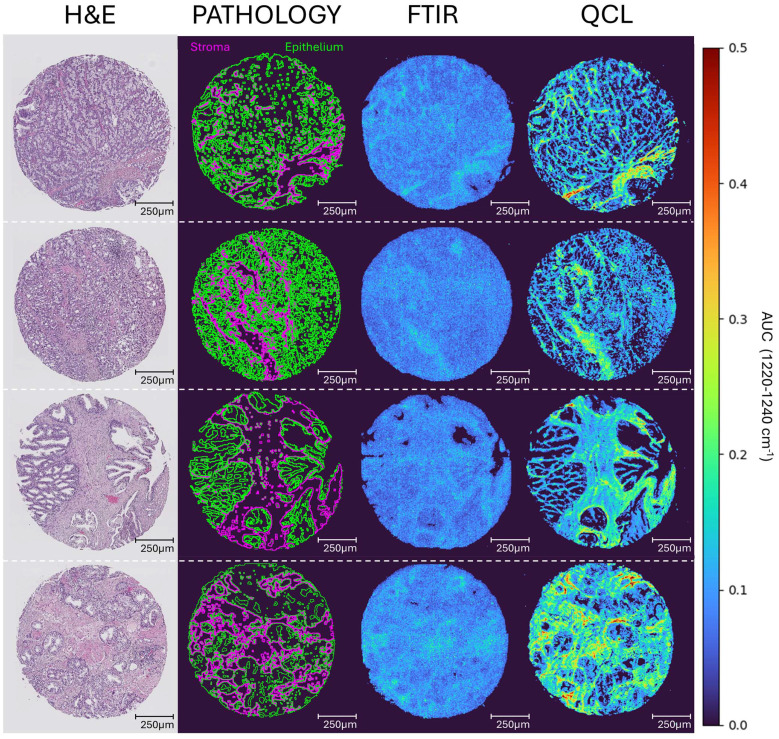
Comparison of integrated lower wavenumber spectral regions (1220–1240 cm^−1^) for unprocessed data of four separate prostate cores imaged using FTIR and QCL systems, with H&E stained adjacent sections and key manually drawn pathological annotations for stroma and epithelial regions (pink and green respectively) provided for visual comparison.

Absorbance plots of randomly selected spectra, as in Fig. 2, help visualize the improved SNR of the acquired data. This is particularly noticeable in the lower wavenumber region of Fig. 2, which contains diagnostically important bands which are nevertheless quite weak compared to the noise. Example bands and their proposed vibrational modes and sources are tabulated in. Comparisons of core images generated from integration of a lower wavenumber region (1220–1240 cm^−1^), as in Fig. 3, show how complex tissue architectures are still being spatially resolved in the QCL data, while being largely obscured in the FTIR data. This underlines that QCL imaging delivers spectral data with better overall SNR, thus, providing for a comprehensive chemical analysis of fine morphological features for which FTIR shows limited applicability.

### Comparing unsupervised clustering applications

**Fig. 4:**
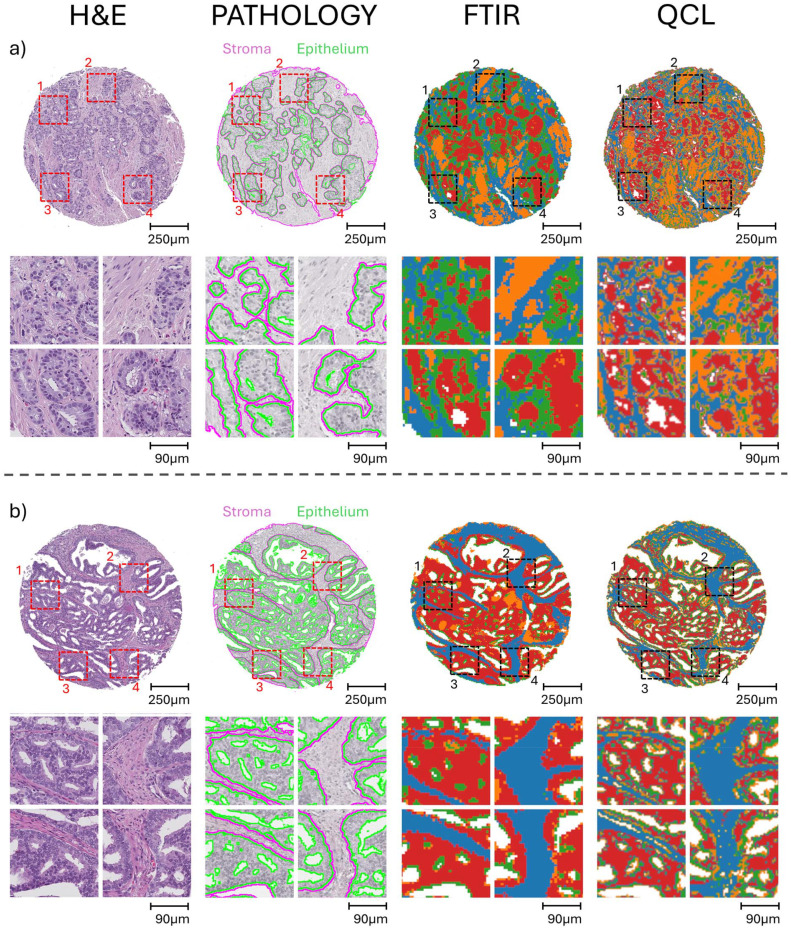
Unsupervised *k*-means clustering images (*n* = 4) for FTIR and QCL hyperspectral images of two cancerous prostate cores (a and b). Clusters are coloured blue, red, green, and orange. H&E stained adjacent sections are provided for each core, with pathological regions showing key stromal and epithelial boundaries (pink and green borders respectively) drawn for interpretation. Four key zoomed in regions of interest (ROI) are presented, with numbers 1 through to 4 corresponding to top left, top right, bottom left, and bottom right ROIs.

In the absence of labelled data, it is still possible to identify and resolve tissue constituents using infrared data, with *k*-means clustering being a commonly used method.^58–61^ Similar chemical fingerprints can be grouped together into a cluster, which when arrayed can create a segmentation map, highlighting key chemical regions within a hyperspectral image. Fig. 4 displays the comparison of clustering results between the FTIR and QCL data for a pair of prostate cores to further highlight differences in data quality. Spectral data for both modalities followed their respective pre-processing treatments and underwent *k*-means clustering with 4 clusters. It can be seen that the FTIR and QCL clusters can overlap substantially in the generalised location of key prostate tissue constituents. However, the cluster results based on the QCL data display an increased level of detail. For instance, the boundaries of identified epithelial regions (red colour) are much more refined in the QCL segmentation map compared to the FTIR (see zoom-ins for Fig. 4a and b). Additionally, it appears that the stromal regions (blue) directly neighbouring the epithelial appears unresolved in the FTIR clusters (see zoom-ins for Fig. 4a and b). Notably, this is not an inherent limitation of FTIR since stroma types have been identified previously, but is a consequence of the reduced number of scans and thus poor SNR dictated by the requirement of reasonable measurement times.^21,62^

### Comparing supervised machine learning applications

**Fig. 5:**
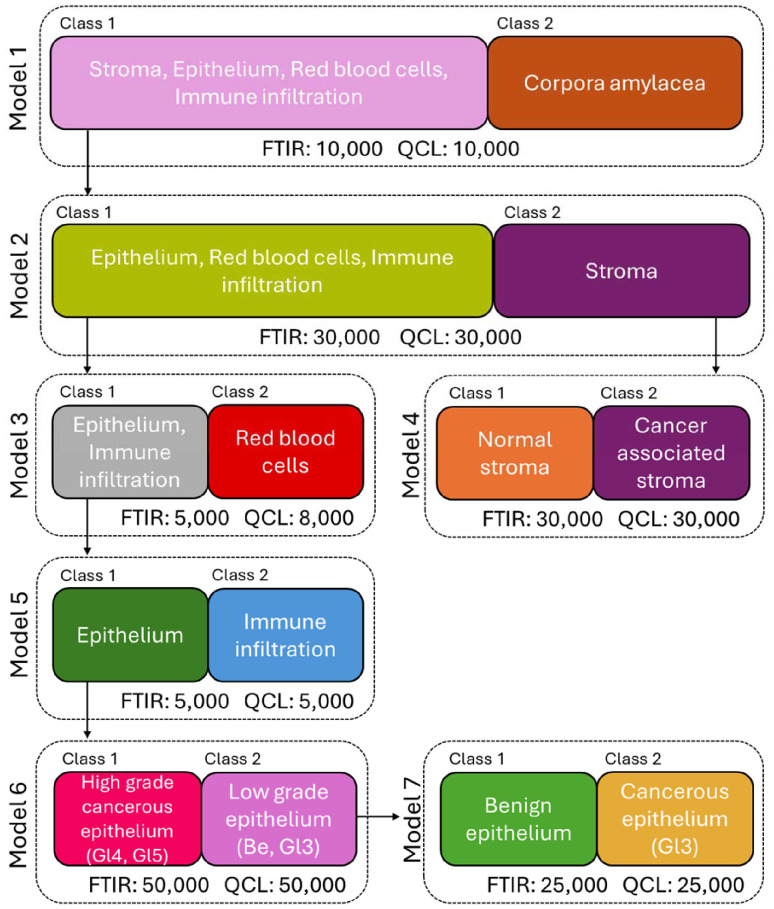
A complex, tiered Random Forests classification architecture covering seven separate models: Model 1 for classifying stroma, epithelium, red blood cells and immune cell infiltration from corpora amylacea; Model 2 for classifying epithelium, red blood cells and immune cell infiltration from stroma; Model 3 for classifying epithelium and immune infiltration from red blood cells; Model 4 for classifying normal and cancer associated stroma; Model 5 for classifying epithelium from immune infiltration; Model 6 for classifying high grade cancerous epithelium (Gleason score 4 and 5) from low grade epithelium (benign and Gleason score 3); Model 7 for classifying benign epithelium and Gleason score 3 epithelium. The number of datapoints for model training for each class in both FTIR and QCL based models are provided.

The performance of supervised machine learning applications can also be compared between QCL and FTIR modalities. This can be done at a simple level where only key tissue constituents are modelled, as well as at a more complex level where subgroupings of several tissue constituents can be modelled. To illustrate, two tiered sets of Random Forests classifiers were built on annotated prostate cancer tissue covering the same patient cores in roughly the same areas, for both simple and complex modelling architectures. The annotations of tissue regions were created under pathologist guidance for 260 tissue cores covering 65 unique patients, and treated as the ground truth for result evaluations. The complex model architecture, including sample sizes for each group, are presented in Fig. 5. The simple model architecture is provided in the ESI (Fig. 1†). Training sets are generated in two steps: First, a main training data set is sampled from all available labelled data for each tissue group to form the training data (in example, 25 000 spectra of benign epithelium). It is from this main group of training data that the individual classes for each model are built. Evenly sized training sets of spectra were randomly selected for each class from the main groups of sampled annotated data, in example, for Class 1 in Model 1 within Fig. 5, each tissue group (stroma, epithelium, red blood cells, and immune infiltration) will have 2500 spectra each, totaling 10 000 spectra for the whole class. This is done to ensure equal representation of tissue groups within classes. Some spectra are used for training in multiple models, however there is no data leakage between training and test sets. All remaining labelled data is used for testing.

In predicting the test data, spectra are passed through the tiered models until a terminal node is reached. In example, for the complex model illustrated in Fig. 5, a spectrum that is predicted as benign epithelium will pass from Model 1 to Model 7 receiving a final prediction label, whereas a spectrum of normal stroma will pass to Model 4 from Model 2. Models were validated *via* 10-fold cross validation, with new models being trained for each fold.

Results of both FTIR and QCL systems are presented as follows: Complex model summary statistics are provided in Table 3, with the fifth ranked fold by model accuracy presented in Table 4. The fifth rank fold is chosen to represent the median model result, resulting in a fairer representation of the model performance. The summary statistics for the simple model results are provided in the ESI (Table 2†) alongside the fifth ranked fold by model accuracy (ESI Table 3†). The calculation of these summary statistics has been discussed in prior works.^26^

**Table 3:** Examples of diagnostically important lower wavenumber absorbance bands. Suggested vibrational modes and source(s) are provided for generic tissue band assignments

Approximate wavenumber (cm^−1^)	Suggested vibrational Mode(s)	Suggested source(s)	Ref.
1030	C–O stretch	Glycogen, collagen	33 and 63–67
1064	C–O ribose stretch	Carbohydrates	68 and 69
1082	Symmetric PO2-stretch	Phosphodiester, nucleic acid	64, 65 and 69–73
1127	C–O stretch	Carbohydrates, sucrose	74
1156	C–O stretch, C–O–C asymmetric stretch	Glycogen, mucin	75
1171	C–OH stretch, CO–O–C asymmetric stretch, CO stretch	Serine, tyrosine, threonine, collagen	63, 70, 72 and 76
1207	Asymmetric PO2-stretch	Phosphates, collagen	67, 69 and 71
1240	C–N stretch, N–H bend, asymmetric PO2-stretch	Proteins (amide III), nucleic acid, collagen	63, 67, 70, 71 and 76–79
1284	N–H bending, C–N stretching	Proteins (amide III), collagen	65, 71 and 80
1317	N–H bending, C–N stretching	Proteins (amide III), collagen	65, 71 and 73
1340	CH2 wagging, C–O stretch	Lipids, collagen	65, 67, 73 and 81
1402	Symmetric CH_3_ bend, C–N stretch, symmetric COO-stretch	Proteins, fatty/amino acids	65, 67 and 82–84

**Table 4:** Summary statistics (sensitivity, specificity, F1 score, precision, and model accuracy) of 10-fold cross validation supervised classification model prediction results for complex models (epithelial and stromal subgroupings) trained and tested on FTIR (top) and QCL (bottom) measured data. Standard deviations of over 0.01 are shown where relevant

	Sensitivity	Specificity	F1 score	Precision
**FTIR 10-fold cross validation average (std dev ≥0.01)**				
Normal epithelium	0.79	0.96	0.84	0.91
Cancerous epithelium (GL3)	0.79 (0.01)	0.96	0.32	0.20
Cancerous epithelium (GL4/5)	0.76	0.96	0.82	0.89
Normal stroma	0.85	0.97	0.88	0.91
Cancerous ass. Stroma	0.84	0.98	0.73	0.65
Immune infiltration	0.90	0.96	0.25 (0.01)	0.14
Red blood cells	0.97	0.99	0.59 (0.02)	0.42 (0.02)
Corpora amylacea	0.99	1.00	0.72 (0.01)	0.57 (0.02)
			**Model accuracy**	**0.80**
**QCL 10-fold cross validation average (std dev ≥0.01)**				
Normal epithelium	0.90	0.97	0.92	0.94
Cancerous epithelium (GL3)	0.90	0.99	0.84	0.78 (0.01)
Cancerous epithelium (GL4/5)	0.90	0.98	0.93	0.97
Normal stroma	0.96	0.98	0.94	0.92
Cancerous ass. Stroma	0.92	0.98	0.86	0.80
Immune infiltration	0.96	0.99	0.68 (0.01)	0.52 (0.01)
Red blood cells	0.97	0.99	0.64 (0.01)	0.48 (0.01)
Corpora amylacea	0.99	0.99	0.81 (0.02)	0.69 (0.02)
			**Model accuracy**	**0.91**

**Fig. 6:**
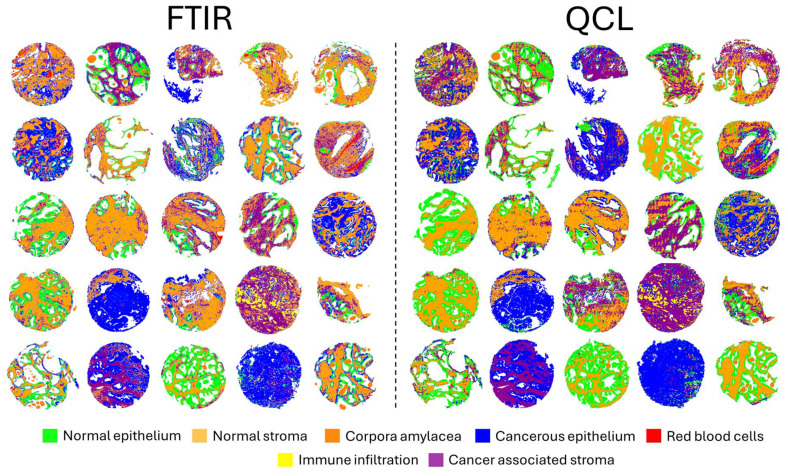
Comparison of the complex multi-tiered supervised classifications of multiple tissue constituents for 25 prostate cores imaged on FTIR (left) and QCL systems (right).

The results obtained highlight the ability to develop extremely well performing models for both modalities when classifying key tissue constituents with sensitivity and specificity values over 0.9 for all classes. For more complex modelling, the QCL system achieves greater results in comparison to the FTIR, maintaining the high sensitivity and specificity values of at least 0.9 across all classes. False colour images can be generated for whole core predictions using the simple and complex models for both FITR and QCL models, as shown in the ESI (Fig. 2†) and Fig. 6 respectively. Predicted cores appear generally consistent in the majority of cases, however some observed differences are expected given the key differences in model performance (Table 5).

**Table 5:** Confusion matrixes of test data prediction results for the best performing model achieved in the 10-fold cross validation of complex models trained on FTIR (top) and QCL (bottom) data. Proportions of total spectra classified in each group is provided in brackets

		Predicted class							
		Normal epith	Canc. epith (Gl3)	Canc. epith (GL4/5)	Normal stroma	Canc. ass. stroma	Immune infiltration	Red blood cells	Corpora amylacea
**FTIR (5** ^**th**^ **rank fold)**									
Actual class	Normal epith	**139 832 (0.79)**	9086 (0.05)	15 149 (0.09)	3264 (0.02)	1434 (0.01)	4049 (0.02)	2532 (0.01)	2047 (0.01)
	Canc. epith (GL3)	377 (0.06)	**4958 (0.78)**	410 (0.06)	68 (0.01)	201 (0.03)	209 (0.03)	43 (0.01)	74 (0.01)
	Canc. epith (GL4/5)	11 777 (0.06)	10 382 (0.06)	**140 734 (0.76)**	1125 (0.01)	4033 (0.02)	15 123 (0.08)	864 (0.00)	558 (0.00)
	Normal stroma	1691 (0.01)	529 (0.00)	517 (0.00)	**119 349 (0.85)**	17 928 (0.13)	325 (0.00)	270 (0.00)	68 (0.00)
	Canc. ass. stroma	216 (0.00)	299 (0.01)	553 (0.01)	6632 (0.13)	**43 382 (0.84)**	289 (0.01)	115 (0.00)	22 (0.00)
	Immune infiltration	65 (0.02)	39 (0.01)	152 (0.04)	57 (0.02)	49 (0.01)	**3379 (0.90)**	15 (0.00)	0 (0.00)
	Red blood cells	13 (0.01)	11 (0.00)	5 (0.00)	16 (0.01)	11 (0.00)	0 (0.00)	**2442 (0.97)**	9 (0.00)
	Corpora amylacea	19 (0.01)	2 (0.00)	0 (0.00)	0 (0.00)	0 (0.00)	0 (0.00)	4 (0.00)	**3727 (0.99)**
**QCL (5** ^**th**^ **rank fold)**									
Actual class	Normal epith	**246 297 (0.89)**	2638 (0.01)	8732 (0.03)	7995 (0.03)	1005 (0.00)	1675 (0.01)	3472 (0.01)	3423 (0.01)
	Canc. epith ( GL3)	804 (0.03)	**21 766 (0.90)**	188 (0.01)	175 (0.01)	827 (0.03)	9 (0.00)	127 (0.01)	271 (0.01)
	Canc. epith (GL4/5)	12 447 (0.04)	3190 (0.01)	**293 679 (0.90)**	1482 (0.00)	8744 (0.03)	2627 (0.01)	2108 (0.01)	1992 (0.01)
	Normal stroma	2332 (0.02)	156 (0.00)	240 (0.00)	**141 107 (0.95)**	3411 (0.02)	117 (0.00)	230 (0.00)	184 (0.00)
	Canc. ass. stroma	140 (0.00)	87 (0.00)	1398 (0.02)	3228 (0.05)	**57 052 (0.92)**	17 (0.00)	104 (0.00)	21 (0.00)
	Immune infiltration	40 (0.01)	18 (0.00)	64 (0.01)	53 (0.01)	24 (0.00)	**4735 (0.96)**	10 (0.00)	1 (0.00)
	Red blood cells	26 (0.00)	12 (0.00)	51 (0.01)	20 (0.00)	16 (0.00)	5 (0.00)	**5410 (0.98)**	2 (0.00)
	Corpora amylacea	63 (0.00)	11 (0.00)	28 (0.00)	0 (0.00)	1 (0.00)	0 (0.00)	2 (0.00)	**12 581 (0.99)**

## Discussion

It is well established that infrared hyperspectral imaging can be used to evaluate tissue constituents and has the potential to be used as a diagnostic tool to aid pathologists. One of the main reasons for the slow progress to clinical adoption is the slow scan times of FTIR for large areas of tissue. QCLs enable the fast scanning of large regions of tissue, but until recently, only if the number of frequencies measured was significantly reduced. This results in potential loss of information and can also be influenced by varying background effects caused by scattering or reflection artefacts.^61,85–90^ Scanning the full fingerprint region to obtain hyperspectral images offered no real advantage over FTIR. However, a new commercially available QCL-based IR microscope allows to perform full fingerprint acquisitions at a rate of 16.8 mm^2^ per minute. Compared to a commercial FTIR microscope, the QCL microscope improves upon the size of the FPA alongside improved nominal pixel size, allowing for larger regions to be scanned with more spectra per area. To highlight the scale of improvement, the collective scan time (excluding instrument calibration and sample purging) needed to image the full set of tissues reported in this publication using the FTIR system was around 180 hours. This time was brought down to 9 hours for the QCL system, a 20-fold improvement. This level of improvement is further increased when considering the time increase needed to perform additional scan co-additions to try and improve the SNR of the FTIR data.

It must also be noted that for many microscopes, there is a further added time for acquisition associated with sample loading and purging of the sample compartment. For microscopes that have a sample compartment it can take upwards of ten minutes per sample to reach a relative humidity close to 0% to remove the effect of water vapour within the spectra, depending on the size of the sample compartment and the flow rate of the purge air. The QCL system used in this study does not have a sample compartment, removing this addition time consideration altogether. Impressively, there does not appear to be any water vapour contribution being detected within the spectral data acquired. There is also the added advantage that the QCL does not need a liquid nitrogen cooled detector and time is saved while not needing to let the detector settle (often 30 minutes after filling) alongside cost savings associated with liquid nitrogen acquisition.

Additional to the improved acquisition time, the SNR of the QCL data is a marked improvement compared to the FTIR data acquired. The better SNR allows smaller absorbing peaks in the lower fingerprint region to be resolved much more easily than in the presence of noise. It is also possible that at a certain SNR, some peaks can be misidentified as being chemical peaks when in fact they are noise, or simply not identified. Furthermore, the impact of noise scaling when calculating the derivatives of spectral data is further reduced with improved SNR. This in turn can lead to improved results for unsupervised techniques such as clustering, where the improved SNR allows for subgroups of tissues (such as loose and dense stromal tissue) to be separated effectively when their differences are small changes to specific peaks (such as collagen). The impact of better SNR data further extends to supervised techniques that form the backbone of diagnostic and prognostic methods relying on the outputs of trained classification algorithms. Lower levels of noise will result in fewer misclassifications, and better performing models. However, with QCL systems the impact of coherence must not be overlooked. In this study the impact of coherence was minimised enough to a level that any coherence artefacts did not meaningfully impact the results of either the unsupervised or supervised approaches applied to the data. It is thought the most impactful reduction of coherence was a combination of instrumentation based coherence reduction, and the preprocessing protocol applied post-acquisition.

Regarding the performance of the supervised classification model obtained in this study, the achieved performance matches that of other models reported in literature, and improves upon the FTIR based model results for complex tissue modelling. The poor F1 score and precision results for classes with high sensitivity and specificity metrics (≥0.90) can be attributed to largely unbalanced sample sizes typically seen in spectroscopic tissue datasets. Assuming no change in sensitivity and specificity, scaling the prediction results such that the total number of datapoints are comparable to the larger datasets (>100 000 spectra) will result in F1 and precision scores >0.9.

While we have shown that the employed QCL microscope does address the acquisition time concern for clinical uptake considerations, there still exists other challenges that must also be mentioned, namely the cost of sample substrates. If this technology is to be used in a routine clinical workflow, the vast number of samples processed in a standard histopathology laboratory would need to be placed on to infrared transmitting substrates for spectroscopic imaging, which becomes exceptionally expensive with sample substrates such as calcium and barium fluoride (CaF_2_ and BaF_2_). One could argue that samples slides could be cleaned for reuse, however this removes the ability to catalogue and archive tissue samples for re-analysis. One way to address this is the use of membrane slides, which are infrared transmitting and a much cheaper alternative to current sample substrates. These slides have been shown to facilitate high quality imaging for QCL systems.^42^

## Conclusions

This study shows that Quantum Cascade Laser systems can acquire high quality full fingerprint region hyperspectral datasets of tissue samples within clinical timeframes, addressing one of the major considerations for clinical uptake of spectroscopic imaging techniques. An entire patient cohort represented by a collection of prostate cancer tissue microarrays were imaged on both QCL and FTIR instruments, with the QCL system achieving better SNR data, higher image contrast resultant of improved nominal pixel size, improved unsupervised clustering of tissues, and comparably high-performing supervised models used for tissue classification, within 1/20^th^ of the time. It is anticipated that this study underlines the potential for QCL spectroscopy to be used within histopathology labs for clinical applications. Future studies will aim to further establish the effectiveness of this novel QCL imaging modality across different tissue types covering various diagnostic and prognostic research questions.

## Author contributions

Dougal Ferguson: conceptualization, methodology, data acquisition, formal analysis, investigation, visualization, writing – original draft, review & editing. Niels Kroeger-Lui: conceptualization, data acquisition, writing – review & editing. Domenic Dreisbach: conceptualization, data acquisition, writing – review & editing. Claire Hart: sample acquisition, sample annotation, pathologist expertise. Diego F. Sanchez: sample annotation, pathologist expertise. Pedro Oliveira: sample annotation, pathologist expertise. Mick Brown: sample acquisition. Noel Clarke: sample acquisition. Ashwin Sachdeva: sample acquisition, supervision, clinical expertise, writing – review & editing. Peter Gardner: conceptualization, writing – review & editing, supervision.

## Ethics approval

The prostate cancer tissue microarray was accessed *via* the Manchester Cancer Research Cancer Biobank (10_NOCL_02) under ethical approval granted by the South Manchester Research Ethics Committee (Ref: 22/NW/0237).

## Data availability

Data used in this paper are held by the primary author, who will share the data upon reasonable request. At the time of submission, discussions are ongoing with the tissue biobank about releasing spectral data through an open repository system such as Zenodo.

## Conflicts of interest

Niels Kroeger-Lui and Domenic Dreisbach are employees of Bruker Optics. All other authors declare no competing interest.

## Supplementary Material

AN-150-D5AN00046G-s001
