# Supplementary material for: Full fingerprint hyperspectral imaging of prostate cancer tissue microarrays within clinical timeframes using quantum cascade laser microscopy
Source: Analyst. 2025 Mar 10;150(9):1741–53. doi: 10.1039/d5an00046g (PMC11907692; doi:10.1039/d5an00046g)
Supplement: AN-150-D5AN00046G-s001 [file AN-150-D5AN00046G-s001.pdf]

This journal is © The Royal Society of Chemistry 2025

Supplementary information: Clinical characteristics

Table 1 – Key clinical characteristics of the prostate cancer tissue cohort.

Age		PSA (ng/mL)		Gleason Group		Tumour stage	
Median (IQR)	74 (10)	Median (IQR)	33 (84.45)	GG1	66 (36.1%)	T1-2	68 (37.1%)
<65 years	25 (13.7%)	0-19.9	63 (34.4%)	GG2-3	49 (26.8%)	T3-4	90 (49.2%)
65-74 years	60 (32.8%)	20-99.9	59 (32.2%)	GG4-5	29 (15.8%)	Missing	25 (13.7%)
>74 years	81 (44.3%)	>=100	37 (20.2%)	Missing	39 (21.3%)		
Missing	17 (9.3%)	Missing	24 (13.1%)				

Supplementary information: Simple model architecture, summary statistics, predictions, and fifth fold results.

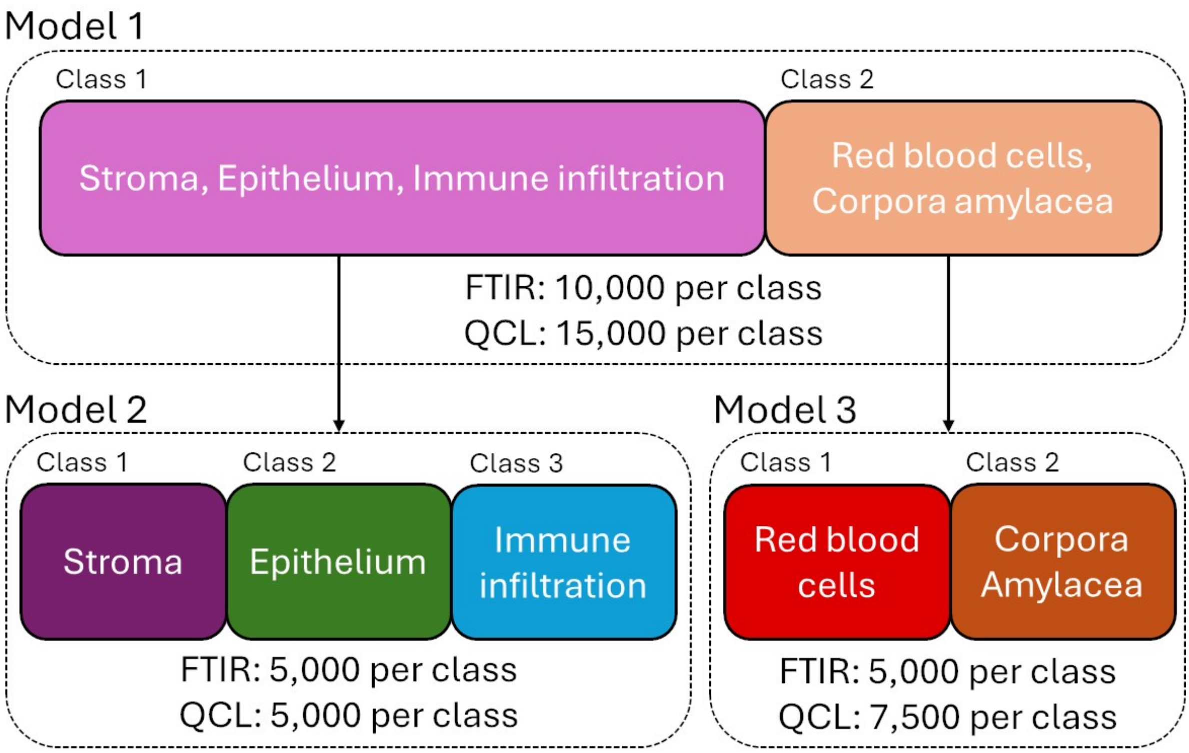

Figure 1 – A simple, tiered Random Forests classification architecture covering three separate models: Model 1 for classifying stroma, epithelium, and immune cell infiltration from red blood cells and corpora amylacea; Model 2 for classifying stroma, epithelium, and immune cell infiltration; Model 3 for classifying red blood cells and corpora amylacea. The number of datapoints for model training for each class in both FTIR and QCL based models are provided.

Table 2 – Summary statistics (sensitivity, specificity, F1 score, precision, and model accuracy) of 10-fold cross validation supervised classification model prediction results for simple models trained and tested on FTIR (top) and QCL (bottom) measured data. Standard deviations of over 0.01 are shown where relevant.

FTIR 10- fold cross validation average (std dev >0.01)

	Sensitivity	Specificity	F1 score	Precision
Epithelium	0.91	0.97	0.95	0.98
Stroma	0.96	0.97	0.96	0.95
Immune infiltration	0.92	0.97	0.22 (0.01)	0.13
Red blood cells	0.97	1.00	0.64 (0.01)	0.48 (0.01)
Corpora amylacea	0.99	1.00	0.85 (0.01)	0.75 (0.01)
			Model accuracy	0.93

QCL 10- fold cross validation average (std dev >0.01)

	Sensitivity	Specificity	F1 score	Precision
Epithelium	0.95	0.97	0.97	0.99
Stroma	0.97	0.97	0.94	0.92
Immune infiltration	0.98	1.00	0.65 (0.01)	0.49 (0.02)
Red blood cells	0.98	0.99	0.60 (0.01)	0.43 (0.01)
Corpora amylacea	0.99	1.00	0.94	0.90 (0.01)
			Model accuracy	0.96

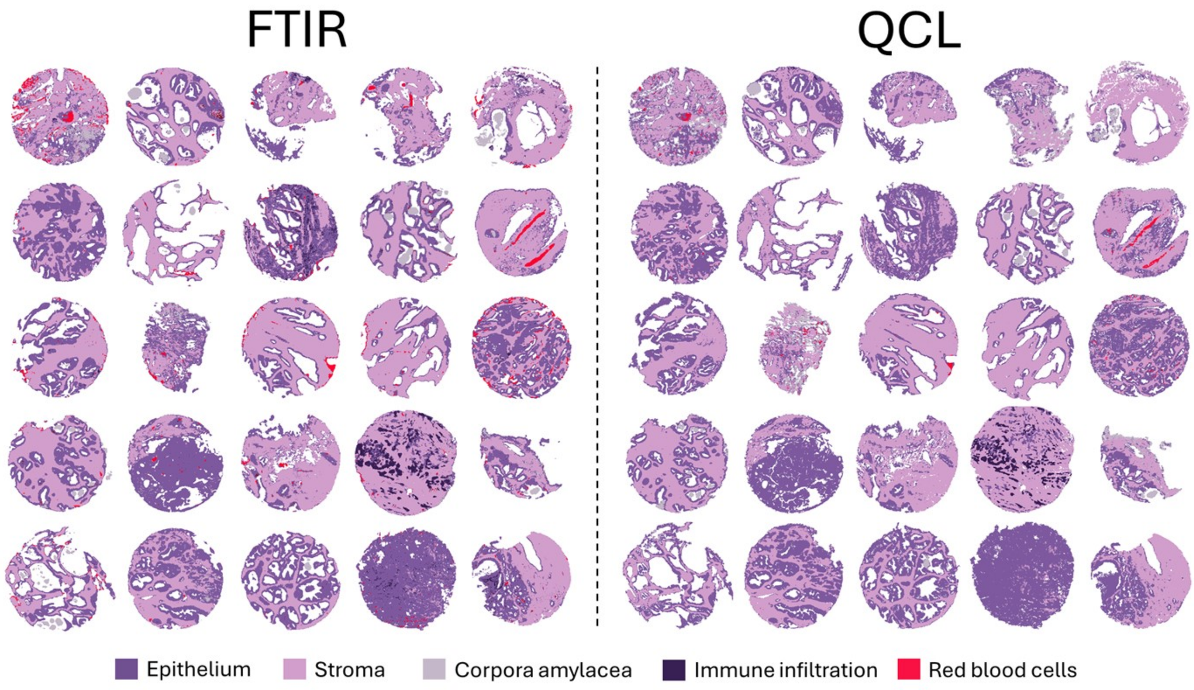

Figure 2 - Comparison of the simple multi-tiered supervised classifications of multiple tissue constituents for 25 prostate cores for FTIR and QCL based models, highlight their similarity in predicting key tissue constituents.

Table 3 – Confusion matrixes of test data prediction results for the best performing model achieved in the 10-fold cross validation of simple models trained on FTIR (top) and QCL (bottom) data. Proportions of total spectra classified in each group is provided in brackets.

FTIR (5th rank fold)

Actual class \ Predicted class	Epithelium	Stroma	Immune infiltration	Red blood cells	Corpora amylacea
Epithelium	**422,861 (0.91)**	12,918 (0.03)	22,554 (0.05)	2,124 (0.00)	2,872 (0.01)
Stroma	7,022 (0.03)	**238,187 (0.96)**	1,371 (0.01)	474 (0.00)	131 (0.00)
Immune infiltration	248 (0.07)	46 (0.01)	**3,458 (0.92)**	4 (0.00)	0 (0.00)
Red blood cells	43 (0.02)	41 (0.02)	0 (0.00)	**2,413 (0.96)**	10 (0.00)
Corpora amylacea	52 (0.01)	1 (0.00)	0 (0.00)	7 (0.00)	**8,692 (0.99)**

QCL (5th rank fold)

Actual class \ Predicted class	Epithelium	Stroma	Immune infiltration	Red blood cells	Corpora amylacea
Epithelium	**685,876 (0.95)**	21,962 (0.03)	3,952 (0.01)	7,334 (0.01)	1,549 (0.00)
Stroma	7,691 (0.03)	**256,376 (0.97)**	403 (0.00)	263 (0.00)	91 (0.00)
Immune infiltration	80 (0.02)	45 (0.01)	**4,818 (0.97)**	2 (0.00)	0 (0.00)
Red blood cells	75 (0.01)	36 (0.01)	8 (0.00)	**5,913 (0.98)**	10 (0.00)
Corpora amylacea	133 (0.01)	6 (0.00)	0 (0.00)	22 (0.00)	**15,025 (0.99)**
